# Navigating an unpredictable environment: the moderating role of perceived environmental unpredictability in the effectiveness of ecological resource scarcity information on pro-environmental behavior

**DOI:** 10.1186/s40359-024-01762-1

**Published:** 2024-05-10

**Authors:** Dian Gu, Jiang Jiang

**Affiliations:** 1 Key Laboratory of Applied Psychology, Chongqing Normal University, Chongqing, China; 2 School of Education, Chongqing Normal University, Chongqing, China; 3 Beijing Key Laboratory of Applied Experimental Psychology, National Demonstration Center for Experimental Psychology Education (Beijing Normal University), Faculty of Psychology, Beijing Normal University, No.19 Xinjiekouwai Street, Beijing, 100875 China

**Keywords:** Ecological resource scarcity information, Environmental unpredictability, Pro-environmental behaviors, Life history theory, Washing-hands paradigm

## Abstract

**Background:**

The global issue of ecological resource scarcity, worsened by climate change, necessitates effective methods to promote resource conservation. One commonly used approach is presenting ecological resource scarcity information. However, the effectiveness of this method remains uncertain, particularly in an unpredictable world. This research aims to examine the role of perceived environmental unpredictability in moderating the impact of ecological resource scarcity information on pro-environmental behavior (PEB).

**Methods:**

We conducted three studies to test our hypothesis on moderation. Study 1 (*N* = 256) measured perceived general environmental unpredictability, perceived resource scarcity and daily PEB frequencies in a cross-sectional survey. Study 2 (*N* = 107) took it a step further by manipulating resource scarcity. Importantly, to increase ecological validity, Study 3 (*N* = 135) manipulated the information on both ecological resource scarcity and nature-related environmental unpredictability, and measured real water and paper consumption using a newly developed washing-hands paradigm.

**Results:**

In Study 1, we discovered that perceived resource scarcity positively predicted PEB, but only when individuals perceive the environment as less unpredictable (interaction effect: 95% *CI* = [-0.09, -0.01], Δ*R*^2^ = 0.018). Furthermore, by manipulating scarcity information, Study 2 revealed that only for individuals with lower levels of environmental unpredictability presenting ecological resource scarcity information could decrease forest resource consumption intention (interaction effect: 95%*CI* = [-0.025, -0.031], Δ*R*^2^ = .04). Moreover, Study 3 found that the negative effect of water resource scarcity information on actual water and (interaction effect: 95%CI = [3.037, 22.097], η_*p*_^2^ = .050) paper saving behaviors (interaction effect: 95%CI = [0.021, 0.275], η_*p*_^2^ = .040), as well as hypothetical forest resource consumption (interaction effect: 95%CI = [-0.053, 0.849], η_*p*_^2^ = .023) emerged only for people who receiving weaker environmental unpredictability information.

**Conclusion:**

Across three studies, we provide evidence to support the moderation hypothesis that environmental unpredictability weakens the positive effect of ecological resource scarcity information on PEB, offering important theoretical and practical implications on the optimal use of resource scarcity to enhance PEB.

**Supplementary Information:**

The online version contains supplementary material available at 10.1186/s40359-024-01762-1.

## Introduction

Ecological resource scarcity, such as water and energy, poses significant challenges in our current times. The reduction of renewable freshwater resources per capita by 55% from 1993 to 2014 emphasizes the urgency of addressing this issue [1]. According to the World Economic Forum (2019), water shortages remain a top concern for policymakers and business leaders worldwide. In response to resource scarcity, various entities, including governments, water utilities, and community-based organizations, have employed different strategies to promote resource conservation [2]. One of the most common approaches is to raise problem awareness by conveying information about resource scarcity [2]. For example, the fact that billions of people lack access to safe water is utilized in the World Water Day campaign in 2023 to encourage more people to take action. Additionally, the Hong Kong SAR Government’s “Let’s Save 10L Water 2.0” campaign emphasizes the importance of conserving water by highlighting the limited availability of this resource.

Despite these efforts, it is important to recognize the complexity and interconnectedness of the world we live in, which makes predicting future environmental conditions challenging. Unforeseen events such as pathogen prevalence, natural disasters, wars, and financial crises illustrate the dynamic nature of our environment. In such an unpredictable world, can simply providing information about ecological resource scarcity lead to a significant increase in pro-environmental behaviors?

In the current research, we aimed to explore whether ecological resource scarcity information could promote pro-environmental behaviors effectively in the unpredictable world. We argued that ecological resource scarcity information is not necessarily useful in promoting pro-environmental behaviors and proposed that environmental unpredictability is a vital factor weakening the effect of ecological resource scarcity on resource consumption.

### Uncertain association between ecological resource scarcity information and pro-environmental behaviors

Based on the information-motivation-behavioral skills (IMB) model, individuals are more likely to change their behavior when they are informed about a problem, along with being motivated to act and have skills to act [3]. In the environmental protection domain, there is a general lack of problem awareness about ecological resource scarcity [4, 5]. This lack of awareness hinders individuals from engaging in pro-environmental behaviors (PEB), which refers to the actions that enhance the quality of the environment, regardless of the intent behind them [6]. Resource conservation campaigns often focus on resource scarcity information to encourage PEB [7]. In some empirical studies, the resource scarcity information was found to be effective. For example, individuals living in regions that experience drought have a higher tendency to make behavioral changes to conserve water [8, 9]. People who perceived stronger ecological resource scarcity reported higher resource-saving behavioral frequencies [10], and indicated a higher frequency of PEB [11]. And water scarcity information was linked to a significant decrease in water use [12–14].

However, we identified some conflicting evidence. Information about resource scarcity is often not sufficient to reduce resource consumption in intervention [15], and the effectiveness of awareness campaigns is unclear [16]. For example, presenting the information about water resource scarcity only was evaluated as ineffective to promote water-saving behaviors by lay people [10]. Energy scarcity information was not strong enough to affect attitudes, intentions, and behaviors toward electricity energy saving [17]. Moreover, resource scarcity information failed to modify resource consumption behaviors in experimental settings [2, 18].

The uncertain relationship between resource scarcity and PEB can be understood through an evolutionary psychological approach. According to the life history theory, individuals may adopt various strategies for allocating resources [19–23]. Those who choose a slow life history strategy prioritize long-term benefits and future planning, which leads them to behave in an environmentally friendly manner for the sake of future generations. On the other hand, individuals adopting a fast life history strategy prioritize immediate gains over long-term consequences [24], resulting in less PEB.

This theory, combined with empirical evidence, suggest that the impact of resource scarcity on PEB may vary depending on the situation, implying that promoting pro-environmental actions may require considering factors beyond simply informing individuals about scarcity. If PEB is seen as an investment in the environment, people engaging in PEB expect long-term benefits from it. However, the environment does not always provide consistent long-term benefits, particularly in today’s unpredictable world. When the expected advantages of environmental protection become uncertain, individuals may prioritize immediate gains, exploit natural resources, and reduce their commitment to PEB. This study hence focuses on the situational factor related to the unpredictable environment, testing its importance in influencing individuals’ PEB under resource scarcity.

### Moderating role of environmental unpredictability

Environmental unpredictability is defined as the level of spatial–temporal variation in environmental harshness [24]. Past empirical studies measured environmental unpredictability in diverse ways [25]. In the current research, we tried to capture both individual-related and nature-related environmental unpredictability in temporal or spatial dimensions. Individual-related environmental unpredictability is mostly indicated by residential changes, and changes in parental financial status for children [19, 24, 26]. It shows whether the structure of an environment, such as the social or economic environment in which one lives, changes over time. Nature-related environmental unpredictability focuses on the pattern of variation that makes environments unpredictable, such as unpredictability of weather and the unpredictability of natural disasters [25].

Based on the life history theory, the environment plays a crucial role in shaping individuals’ life history strategies [19–23]. In predictable environments individuals are more likely to adopt a slow life-history strategy, while highly unpredictable environments promote a fast life-history strategy [24]. Importantly, environmental unpredictability during childhood can influence short-sighted tendencies [27–30], and this effect can also be observed in adulthood [31]. In an unpredictable environment, individuals prioritize immediate desires over future needs because investing in long-term environmental protection may not yield future benefits. This has implications for PEB, as present efforts on environmental protection may not be effective in improving resource scarcity in the future when the environment is unpredictable.

There are two aspects that illustrate the expectation that PEB efforts may not pay off in unpredictable environments. Firstly, in an unpredictable environment, there is a flow of uncontrollable information, which makes it challenging for individuals to maintain strong beliefs that their actions can bring about positive outcomes, such as improving resource scarcity [32]. According to the theories of reasoned action and planned behavior, the impact of awareness of the problem on behavior is greater when individuals perceive a higher level of control over their actions [33]. Hence, environmental unpredictability not only reduces the perceived personal control but also creates a barrier between scarcity awareness and PEB.

Secondly, in unpredictable environments, individuals are more likely to fear free riders, which further hinders behavioral change towards environmental protection under resource scarcity. When deciding whether to take action to protect the environment, people consider whether others will cooperate. However, in unpredictable environments, the likelihood of others investing in PEB becomes uncertain as well, which induces a heightened fear of free riders. For instance, experimental games have shown that individuals behave less cooperatively and invest fewer public goods when the probability of benefiting from them is uncertain [34]. Moreover, studies have demonstrated that individuals are less likely to prioritize the interests of others over their own when environmental unpredictability is primed [31, 35]. Due to the fear that others will not take action in an unpredictable environment, individual efforts to protect the environment may appear less effective in solving the issue of resource scarcity.

Taken together, stronger environmental unpredictability is associated with a fast life-history strategy characterized by low self-efficacy and high fear of free riders, which ultimately leads to less PEB performance in the face of resource scarcity. Both multilevel and individual-level studies have indicated that psychological traits similar to the fast life history strategy weaken the association between environmental problem awareness and actual PEB [10, 36]. Besides, some indirect evidence revealed that resource scarcity and environmental unpredictability could lead to some psychological outcomes that go against promoting PEB. Specifically, poorer childhood and economic uncertainty jointly increase the present orientation and decrease the sense of control [37, 38]. A strong present orientation and low sense of control discourage people from taking actions to save resources [39]. With the above in mind, the following moderation hypothesis was proposed:

Hypothesis: Environmental unpredictability will moderate the effect of ecological resource scarcity on PEB. Specifically, ecological resource scarcity information would play a less effective role in promoting PEB when environmental unpredictability is stronger.

### Current research

In the current research, we conducted three studies to test our hypothesis on moderation. In Study 1, we examined whether perceived general environmental unpredictability would moderate the relationship between perceived resource scarcity and daily PEB frequencies. Study 2 took it a step further by manipulating resource scarcity to test whether the positive effect of ecological resource scarcity information on forest resource consumption intention would be weakened by individual-related environmental unpredictability, specifically the frequency of residential changes. Importantly, to increase ecological validity, Study 3 manipulated the information on both ecological resource scarcity and nature-related environmental unpredictability, and measured real water and paper consumption using a newly developed washing-hands paradigm.

## Study 1

To examine the moderating effect of environmental unpredictability on the relationship between ecological resource scarcity and daily PEB frequency, we conducted a cross-sectional survey for Study 1. We hypothesised that ecological resource scarcity would predict higher frequencies of daily PEB for individuals who perceived the environment as predictable. However, we expected this positive association to diminish for individuals who perceived high levels of environmental unpredictability.

### Participants

To ensure sufficient statistical power (80% power, α = .05) to detect a small-to-medium-sized effect for our moderation hypothesis, based on previous research in the same domain [10], we estimated that a sample size of 256 participants would be required using G*Power 3.1 [40]. Participants were recruited from a Chinese online survey platform (www.wjx.cn) and received monetary compensation for their participation. The survey platform utilized a voluntary opt-in panel, inviting users to complete the questionnaire. A total of 263 participants from China completed the survey. It is important to note that data collection was planned to conclude once 256 observations were collected within a three-week period.

The average age of the participants was 32.21 ± 7.11 years (ranging from 18 to 66 years), with 44.1% of them being male (*N* = 116). In terms of educational attainment, 1.9% held a middle-school degree or below, 1.9% had a high school degree, 8.7% held a junior college degree, 79.8% had a bachelor’s degree, and 7.6% had a master’s degree or higher. The average annual family income was 23.65 ± 21.04 ten thousand yuan.

### Procedure and measures

To address the potential influence of priming participants’ perceived resource scarcity through items expressing the seriousness of resource scarcity [11, 41, 42], we carefully structured the data collection process. Firstly, we measured the dependent variable, PEB frequencies. Following this, participants completed the measure of perceived environmental unpredictability, and subsequently rated their perceived ecological resource scarcity. Additionally, to account for potential bias in self-reported PEB due to social desirability [43], we included a measurement of social desirability as a control variable. Finally, participants provided their demographic information, including age, gender, educational attainment, and annual personal income.

#### Perceived resource scarcity

The measurement of perceived ecological resource scarcity, consisting of 5 items, was adapted from a previous study conducted by Gu and her colleagues [10] (Cronbach’s *α* in the current study is 0.79). Participants were asked to indicate their level of agreement with statements such as “There are not enough resources for everyone in the place where I live” and “In the place where I live, I have already noticed some signs of resource scarcity.” Each item was rated on a 7-point Likert scale, ranging from 1 (*strongly disagree*) to 7 (*strongly agree*). The mean score of the entire scale was computed. Higher scores on this scale indicated higher levels of perceived ecological resource scarcity.

#### Perceived general environmental unpredictability

The item “For me, the environment we live in is unpredictable” developed by Reynolds and McCrea [44], was used to measure how participants perceived the general unpredictability of their environment. Participants rated this item on a 7-point Likert scale, ranging from 1 (*strongly disagree*) to 7 (*strongly agree*). Higher score indicated stronger perceived unpredictability.

#### Daily PEB frequency

Participants were asked to rate the frequency of PEB in their daily lives on a scale from 1 (*never*) to 5 (*always*). They were presented with six common resource conservation actions and asked to consider their behaviors in the year prior to the survey. The items are “do not turn the tap to the maximum when using water”, “switch off the lights when you leave”, “set the air conditioner’s temperature to 26–28 degrees centigrade in summer”, “buy and use energy-efficient appliances”, “avoid using disposable tableware whenever possible”. These six PEB were then converted into a PEB frequency scale, and a mean score was calculated for each participant. Higher scores indicated a higher frequency of PEB. Although the Cronbach’s α for the PEB scale was relatively low at .50, we decided to keep the measure because the items were face-valid. It is worth noting that removing any of the items did not improve the Cronbach’s alpha. Consistent with findings from previous studies, different types of PEB were not completely consistent [45, 46]. And importantly, using the common score derived from the six items did not significantly alter the results.

#### Social desirability

Social desirability was measured using the liar subscale of the Eysenck Personality Questionnaire (EPQ) [47]. This subscale consists of 12 items, with participants answering each question with a “*Yes*” or “*No*” response. A code of 1 was assigned to “*Yes*” and 0 to “*No*”. Higher scores on this subscale indicated a stronger tendency towards social desirability. The measure demonstrated good internal consistency with a Cronbach’s *α* of .75.

### Results

#### Correlation analyses

Prior to conducting hypothesis testing, all variables exhibited normal distributions, as indicated by skewness values ranging from -0.89 to + 0.05 and kurtosis values ranging from -0.72 to + 0.77. We computed Pearson’s correlation coefficients to explore the relations among the studied variables (see Table 1 for descriptive statistics and intercorrelation coefficients). We found a marginally significant positive relationship between perceived ecological resource scarcity and PEB frequency (*r* = 0.12, *p* = .058). And there was no correlation between environmental unpredictability and PEB frequency (*r* = -0.07, *p* = .29). Importantly, as expected, social desirability was positively associated with PEB (*r* = 0.30, *p* < .001), indicating that it should be controlled for in subsequent analyses.

**Table 1:** Descriptive statistics and intercorrelations between variables in study 1

	*M*	*SD*	1	2	3	4	5	6	7	8
1. Gender	-	-	-							
2. Age	32.21	7.11	0.17^**^	-						
3. Educational level	4.89	0.63	-0.04	-0.29^***^	-					
4. Annual income	23.65	21.04	-0.09	-0.04	0.09	-				
5. Social desirability	5.78	2.89	-0.18^**^	0.09	0.06	0.10	-			
6. Perceived ecological resource scarcity	5.08	0.93	-0.01	0.05	-0.01	-0.02	0.04	-		
7. Environmental unpredictability	4.67	1.57	-0.03	-0.12^*^	-0.06	-0.03	-0.21^**^	0.09	-	
8. PEB frequencies	3.57	0.54	0.05	0.01	0.15^*^	-0.03	0.30^***^	0.12^a^	-0.07	-

^a^*p* = .058

^*^p < .05

^**^p < .01

^***^p < .001

#### Moderation analyses

To examine the impact of environmental unpredictability on the relationship between perceived ecological resource scarcity and PEB, we used the PROCESS macro for SPSS [48]. Controlling for social desirability, we found a significant interaction effect between perceived ecological resource scarcity and environmental unpredictability (*b* = -0.05, *SE* = 0.02, *t* = -2.26, *p* = .025, 95% *CI* = [-0.09, -0.01], Δ*R*^2^ = 0.018). To further understand this interaction, we conducted a floodlight analysis [49]. The results showed that perceived ecological resource scarcity was positively and significantly associated with PEB when environmental unpredictability was below 4.41 (*b* = 0.07, *SE* = 0.03, *t* = 1.97, *p* = .05, 95% *CI* = [0.000, 0.136]), but not when it was above 4.41.

**Fig. 1:**
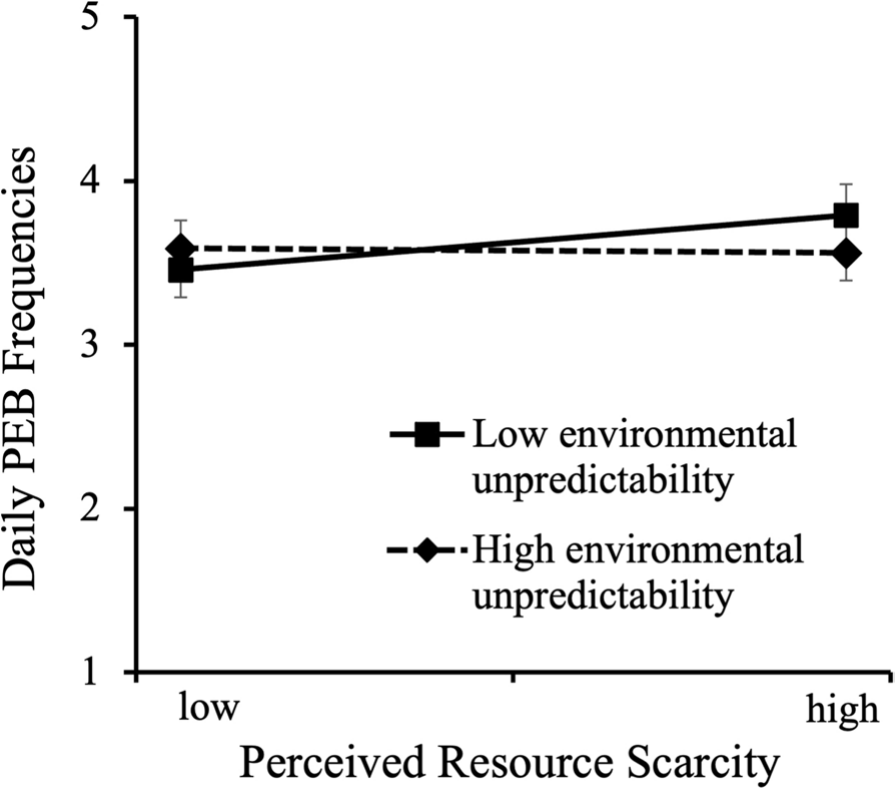
The effect of resource scarcity on PEB at different levels of environmental unpredictability (Study 1)

Additionally, we performed a simple slope analysis to examine the relationship between perceived ecological resource scarcity and PEB for individuals with different levels of perceived environmental unpredictability with social desirability controlled (see Fig. 1). The results indicated that perceived ecological resource scarcity positively predicted PEB for individuals with lower levels of environmental unpredictability (-1 *SD*), *b* = 0.13, *SE* = 0.05, *t* = 2.83, *p* = .005, 95% *CI* = [0.039, 0.219]. However, this relationship was not significant for individuals with higher levels of environmental unpredictability (+ 1 *SD*), (*b* = -0.02, *SE* = 0.05, *t* = -0.35, *p* = 0.73, 95% *CI* = [-0.114, 0.079]).

Furthermore, controlling for demographic variables did not significantly change the results of moderation analysis. In summary, individuals who perceived the environment as more predictable were more likely to engage in PEB when facing ecological resource scarcity.

### Brief discussion

Study 1 identified a moderating effect of environmental unpredictability on associations between perceived ecological resource scarcity and daily PEB. Individuals who perceived the environment as less unpredictable were more likely to adopt environmentally friendly ways to respond to ecological resource scarcity. However, it is important to consider the potential influence of responding to the PEB items on participants’ perceptions of ecological resource scarcity. The act of responding to these items may have directed participants’ attention towards environmental issues, potentially leading to an implicit increase in their perceived ecological resource scarcity. Therefore, it is not possible to infer the direction of the causal relationship between perceived ecological resource scarcity and PEB frequencies solely from correlational data. In addition, using a single item for measuring environmental unpredictability may raise concerns about the comprehensiveness of measurement. To address these limitations, we conducted Study 2, where we manipulated perceived ecological resource scarcity in order to demonstrate its causal effect, and further explore the moderating effect of environmental unpredictability by using another measurement.

Furthermore, it is important to note that the observed moderation effect size was small, which could be attributed to the fact that we measured various types of PEB in this study. According to the Goal System Theory, PEB can be motivated by multiple goals. In the context of resource scarcity, individuals who perceive the environment as more predictable are more likely to prioritize environmental protection for the benefit of future generations, especially if they themselves also stand to gain [50]. For instance, engaging in electricity-saving behaviors not only benefits the environment in the long run but also reduces personal electricity bills. In other words, personal benefits may matter. In our subsequent studies, we will focus on examining PEB that does not involve salient personal benefits in order to highlight the moderating effect of environmental unpredictability.

## Study 2

In Study 2, we sought to replicate the moderating effect of environmental unpredictability on the link between ecological resource scarcity and PEB by manipulating resource scarcity information. We proposed that receiving ecological resource scarcity information would increase PEB intention for individuals with lower levels of environmental unpredictability but that the effect would disappear for individuals with higher levels of environmental unpredictability.

### Participants

To test our moderation hypothesis, we determined that a sample size of 107 would be necessary to achieve 80% power (α = .05) in order to detect a small-to-medium-size effect (*f*^2^ = .075) based on previous research [10] using G*Power 3.1 [40]. We established the rule for ending data collection prior to gathering data, stipulating that the survey link would be closed after obtaining more than 150 observations. Ultimately, we recruited 155 Chinese adults who completed an anonymous online questionnaire and all of these responses were valid.

The participants had an average age of 32.91 ± 10.10 years (range = 18–59 years) and 41.90% of them were males (*N* = 65). In terms of educational attainment, 9.70% held a high school degree, 16.1% held a junior college degree, 66.6% held a bachelor’s degree, and 13.5% held a master’s degree or higher. The average annual personal income was 11.18 ± 44.58 ten thousand yuan.

### Procedure and measures

In the present study, participants reported their demographic information first. Then, environmental unpredictability was measured. Next, participants were randomly assigned to one of two experimental conditions to read a news article, where exposure to the information of resource scarcity (vs. control condition) was the manipulated factor. Finally, PEB intention was measured using a forest management task.

### Manipulation of ecological resource scarcity information

Participants were assigned at random to read one of two news articles. The articles were created specifically to manipulate perceptions of ecological resource scarcity. In the scarcity group (*n* = 77), participants read an article titled “Interpretation of China’s Resources through Big Data: Invisible Resource Scarcity in China”, which highlighted the severity of natural resource scarcity in China. In the control group (*n* = 78), participants read an article of similar length that aimed to evoke similar levels of negative arousal. This article was titled “Interpretation of Sleep through Big Data: Invisible Sleeping Problems in China” and discussed sleep issues in China. To ensure the credibility of the mock news articles, participants were informed that the articles were sourced from *The People’s Daily*, a reputable Chinese newspaper.

Immediately after reading their respective article, participants rated their perception of ecological resource scarcity using a 7-point Likert scale ranging from “*strongly disagree*” (1) to “*strongly agree*” (7). The item presented was: “Currently, I believe that we live in an environment where natural resources are extremely scarce.” Besides, participants also responded to one item on their mood at the moment for the manipulation check on a 7-point Likert scale (1 = “*very negative*” to 7 = “*very positive*”).

#### Environmental unpredictability

At the individual level, environmental unpredictability is mostly indicated by residential changes [24, 25]. The frequency of residential changes showed whether the structure of an environment one lives in changes over time, which is the important aspect of environmental unpredictability. Therefore, Study 2 used the frequency individuals moved in the past to represent their environmental unpredictability. Higher score indicates stronger environmental unpredictability (*M* = 3.59, *SD* = 2.17, *Min* = 0, *Max* = 11). The variable showed approximately normal distribution, with skewness = 0.64 and kurtosis = 0.65. Hence, the raw data of moving frequency are used for analysis.

#### PEB intention

A forest management task was used to measure PEB intention, specifically in relation to forest resource conservation intention [51]. Participants were asked to imagine that they were the owner of a timber company and must compete with three other companies to harvest timber in the same forest. They need to cut down as many trees as possible for their companies to profit and thrive. However, the rapid deforestation could lead to forest destruction. Then, participants were asked to answer one question about deforestation rate on a 7-point Likert scale, ranging from 1 (*very slow*) to 7 (*very fast*), which asked, ‘How fast do you want your company to cut down trees?’ Additionally, they were asked one question about forest resource consumption, ranging from 1 to 100 acres, which asked, ‘How many acres of trees do you expect your company to cut down?’. Give that both questions indicate greedy for forest resources, the average of participants’ reversed standardized scores on the two questions was computed to represent PEB intention. Higher scores indicate stronger forest resource conservation intention. We also treated the two items separately to test our hypothesis, which can be found in the Additional file 1.

### Results

#### Manipulation checks

The manipulation of resource scarcity information was successful. Specifically, participants in the *scarcity* condition (*M* = 5.17, *SD* = 1.25) compared to those in the *control* condition (*M* = 4.55, *SD* = 1.56), reported higher levels of awareness on ecological resource scarcity, *t*(153) = 2.72, *p* = .007, 95%CI = [0.169, 1.066], *d* = 0.44. Furthermore, there was no difference of mood between the two conditions (*M*_scarcity_ = 5.06, *SD*_scarcity_ = 1.19; *M*_control_ = 4.92, *SD*_control_ = 1.23), *t*(153) = 0.73, *p* > .05, 95%CI = [-0.526, 0.242].

#### Hypothesis test

To test for the moderating effect of environmental unpredictability, we regressed the forest resource conservation intention on ecological resource scarcity information (dummy coded: 1 = *scarcity* condition, 0 = *control* condition), environmental unpredictability and their interaction by employing the PROCESS macro (Model 1, 5000 bootstrap samples) for SPSS [48]. The results showed a significant main effect of ecological resource scarcity information (*b* = 0.63, *SE* = 0.23, *t* = 2.78, *p* = .006, 95%*CI* = [0.183, 1.078]). And there was no main effect of environmental unpredictability (*b* = 0.04, *SE* = .03, *t* = 1.23, *p* > .05, 95%*CI* = [-0.026, 0.109]).

**Fig. 2:**
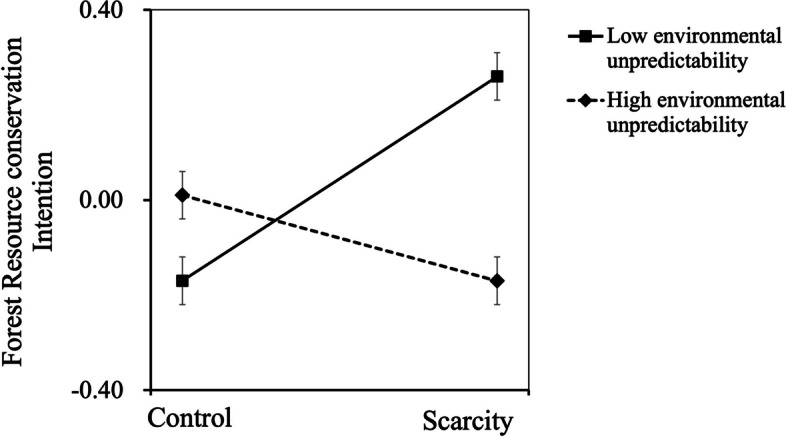
The effect of resource scarcity × environmental unpredictability on forest resource conservation intention (Study 2)

Results showed a significant interaction effect (*b* = -0.14, *SE* = 0.06, *t* = -2.54, *p* = .012, 95%*CI* = [-0.025, -0.031], Δ*R*^2^ = .04), meaning that the effect of ecological resource scarcity information on forest resource conservation intention was moderated by environmental unpredictability. Specifically, for individuals with lower levels of environmental unpredictability (below 1 *SD*), participants in the *scarcity* condition exhibited stronger forest resource conservation intention relative to those in the *control* condition, *b* = 0.43, *SE* = 0.16, *t* = 2.63, *p* = .0095, 95% CI = [0.107, 0.755]. In contrast, for individuals with higher levels of environmental unpredictability (above 1 *SD*), the ecological resource scarcity manipulation had no effect on forest resource conservation intention, *b* = -0.17, *SE* = 0.17, *t* = -1.04, *p* > .05, 95% CI = [-0.512, 0.158] (see Fig. 2).

Besides, a floodlight analysis was performed to decompose the interaction [49]. It revealed that ecological resource scarcity manipulation increased forest resource conservation intention for any value of environmental unpredictability less than 2.78 (*b* = 0.24, *SE* = 0.12, *t* = 1.98, *p* = .05, 95% CI = [0.000, 0.487]), but not for any value greater than 2.78. More importantly, the above findings did not significantly differ after controlling for demographic variables.

### Brief discussion

Study 2 replicated results of Study 1 and identified that environmental unpredictability weakened the positive effect of ecological resource scarcity information on resource conservation. Presenting ecological resource scarcity information could effectively increase forest conservation intention, particularly for individuals who move less frequently, indicating lower levels of environmental unpredictability.

However, the results of Study 2 were limited in several aspects. First, environmental unpredictability can be caused either by individuals themselves, such as frequent relocation, or by nature, such as unforeseen natural disasters. The present study focused on individual-related environmental unpredictability only. Secondly, the measurement of resource conservation intention instead of actual behaviors may have restricted the ecological validity of the findings. Thirdly, it is possible that the moderation effect was underestimated. In the forest management task, the psychological experience of forest resource scarcity may have been primed in both conditions, as participants were informed about the need to compete with other companies for limited forest resources. Consequently, participants’ decisions may have been heavily influenced by the forest management scenario.

## Study 3

Based on above discussions of Study 2, in Study 3, actual PEB was measured to increase ecological validity, and nature-caused environmental unpredictability was focused to improve generalizability. In addition, hypothetical forest resource conservation was also measured to replicate findings of Study 2. We proposed that receiving ecological resource scarcity information would increase actual resource conservation and forest resource conservation intention under predictable environmental conditions but that this effect would disappear under unpredictable environmental conditions.

### Participants

We conducted a power analysis through G*Power 3.1 with the moderating effect size in Study 2, which suggested that a sample size of 135 would be required to achieve 80% power (*α* = .05) [40]. A total of 142 college students in Beijing, China was recruited to participate in the experiment in exchange for monetary compensation. Six participants who failed to finish all experimental tasks were excluded from data analysis. It is worth noting that the rule for terminating data collection was decided before data collection began: the experiment was terminated when more than 135 observations were collected in two weeks.

The average age of the participants was 21.87 ± 2.67 years (range = 17–29 years), and 75.00% of them were female (*N* = 102). The average annual household income was 12.37 ± 17.10 thousand *yuan*.

### Research design and procedure

A 2 (water resource scarcity vs. control) × 2 (unpredictable vs. predictable environment) between-subject design was used.

Before arriving at the lab, participants were asked to fill out their demographic information in an online survey. Upon arrival at the lab, participants were randomly assigned into one of four groups to read a newspaper. These newspapers were designed to be looked like real *Beijing Daily* newspapers. In each type of newspaper, there were two pieces of news. One was designed to manipulate the water resource scarcity information, and another was designed to manipulate environmental unpredictability information. Then, actual water and paper consumption data was recorded in a washing-hands paradigm. Finally, forest resource consumption intention was measured.

### Materials

#### Manipulation of water resource scarcity information

Similar to Study 2, in the *scarcity* condition (*n* = 67), the news article described the seriousness of water resource scarcity in Beijing. While, in the *control* condition (*n* = 69), the news article described Beijing residents’ sleep problems. After reading the article, participants responded to 1 item on perceived ecological resource scarcity on a 7-point Likert scale (1 = “*strongly disagree*” to 7 = “*strongly agree*”), which was adapted from new ecological paradigm scale (NEP): “The earth has plenty of natural resources if we just learn how to develop them” [52].

#### Manipulation of environmental unpredictability information

In the *unpredictable* condition (*n* = 68), the news article was titled “Natural Disasters are Unpredictable and Difficult to Prevent: 9.578 million People were Affected by Various Natural Disasters in January”. The news conveyed the information that natural disasters happened frequently, which caused many people to be affected in January, and there was no way to predict and prevent disasters. By contrast, in the *predictable* condition (*n* = 68), the news stated that even though natural disasters are frequent in China and many people were affected, now some devices can help predict and prevent disasters. The title was “Predication and Prevention of the Occurrence of Natural Disasters is Possible: 9.578 million People were Affected by Various Natural Disasters in January”.

Manipulation check items were rated right after reading the news article. Participants responded to 2 items about perceived unpredictability on a 7-point Likert scale (1 = “*strongly disagree*” to 7 = “*strongly agree*”): “The environment where I live is unstable”, and “The environment where I live is unpredictable”. The average score of the two items was computed such that a higher score indicated stronger perceived unpredictability.

#### Actual water and paper resource consumption

To cover up our real purpose, participants were told that the research was attempting to study palms, so that we would collect their fingerprints in the study. In the washing-hands paradigm, participants were asked to use the inkpad and leave their fingerprints on a sheet of white paper to study their palms. After that, they had to wash their hands in the lab. The amount of water and paper they used was recorded.

**Fig. 3:**
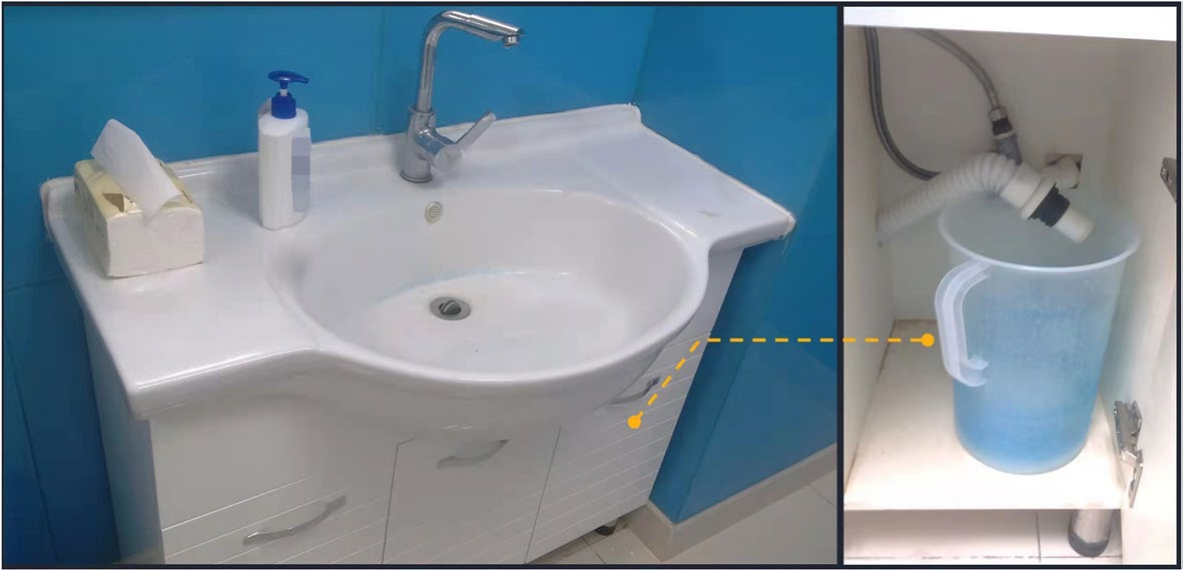
Set-up of washing-hands paradigm

To measure the water consumption, the experimenter placed one measuring cylinder under the washbasin, and the measuring cylinder was linked to the washbasin’s outlet pipe. Importantly, participants could not see the cylinder. To measure their paper consumption, a bag of paper was placed on the washbasin for the participants to use. Besides, to exclude the experimenter effect, participants washed their hands without experimenter observation. Importantly, participants did not know that their behaviors were recorded, and participants were not aware of the real purpose of the study (see Fig. 3). All of the participants were debriefed at the end of the study.

Considering that water and paper consumption for washing ink from hands might be affected by palm size, we recorded the palm area for each participant based on their fingerprints. Then, actual resource consumption was represented by average water consumption and average paper consumption, calculated by water or paper consumption divided by palm area.

#### Hypothetical forest resource conservation

Same as Study 2, the forest management task was used. After reading the scenario, participants were asked to answer the question, “How many acres of trees do you expect your company to cut down?”, ranging from 1 to 100 acres. A higher score on the measurement indicates a lower intention for forest resource conservation.

### Results

#### Manipulation checks

Perceived resource scarcity was significantly greater in the *scarcity* condition (*n* = 67, *M* = 5.61, *SD* = 1.19) than that in the *control* condition (*n* = 69, *M* = 5.13, *SD* = 1.38), *t*(134) = 2.17, *p* = .032, 95%CI = [0.043, 0.920], *d* = 0.37. Perceived unpredictability was also significantly greater in the *unpredictable* condition (*n* = 68, *M* = 5.13, *SD* = 1.28) compared to the *predictable* condition (*n* = 68, *M* = 4.55, *SD* = 1.39), *t*(134) = 2.51, *p* = .013, 95%CI = [0.121, 1.026], *d* = 0.43. Overall, the manipulations were successful and valid.

#### Hypothesis test

To examine the interaction effect between water resource scarcity and environmental unpredictability on resource conservation, two-factor MANOVAs were conducted.

**Fig. 4:**
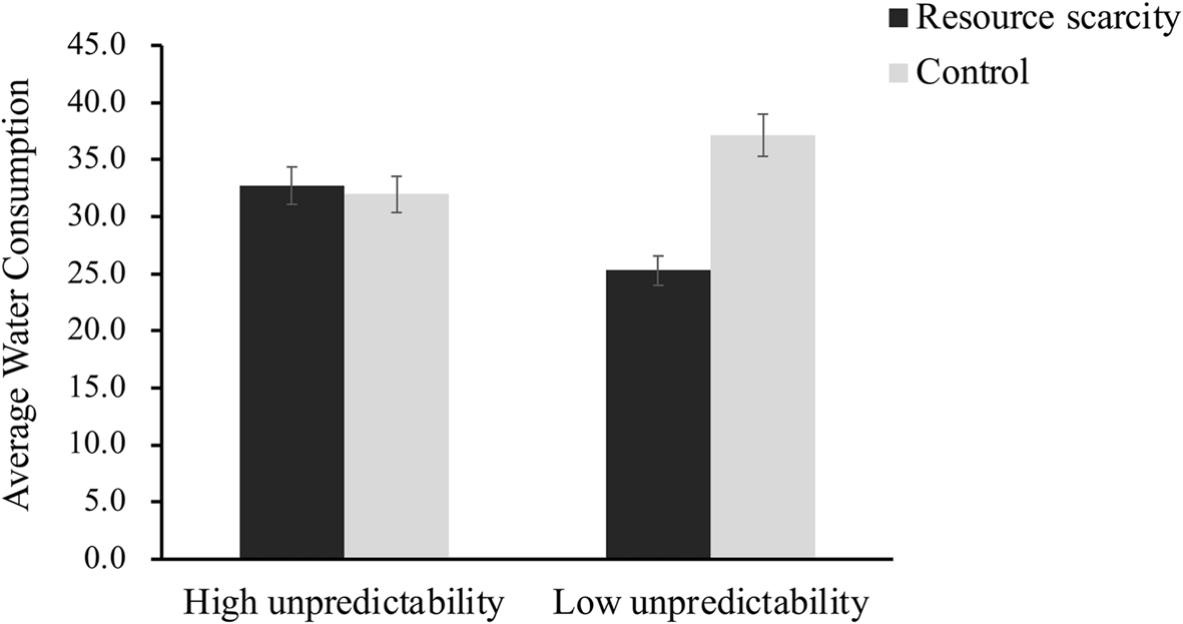
Average water consumption as a function of resource scarcity and environmental unpredictability manipulations (Study 3)

Concerning the average water consumption, gender, age, household income, and cleanliness habits are included as control variables. The findings revealed that the main effect of scarcity was significant (*F*(1,128) = 5.44, *p* = 0.021, 95%CI = [-14.168, -0.673], η_*p*_^2^ = .041), and the main effect of environmental unpredictability was not significant (*F*(1,128) = 0.23, *p* > .05, 95%CI = [-7.437, 5.984]). As expected, the interaction was significant (*F*(1,128) = 6.81, *p* = .01, 95%CI = [3.037, 22.097], η_*p*_^2^ = .050). Then, simple effect analysis revealed that under the *predictable* condition, the average water consumption was significantly less under the *scarcity* condition (*M* = 25.29, *SD* = 13.87) than under the *control* condition (*M* = 37.13, *SD* = 13.91), *F*(1,128) = 12.25, *p* < .001, 95%CI = [-18.535, -5.146], η_*p*_^2^ = .087. However, under the *unpredictable* condition, there was no significant difference between the *scarcity* condition (*M* = 32.71, *SD* = 13.93) and *control* condition (*M* = 31.98, *SD* = 13.90), *F*(1,128) = 0.05, *p* > .05, 95%CI = [-5.984, 7.437] (see Fig. 4).

**Fig. 5:**
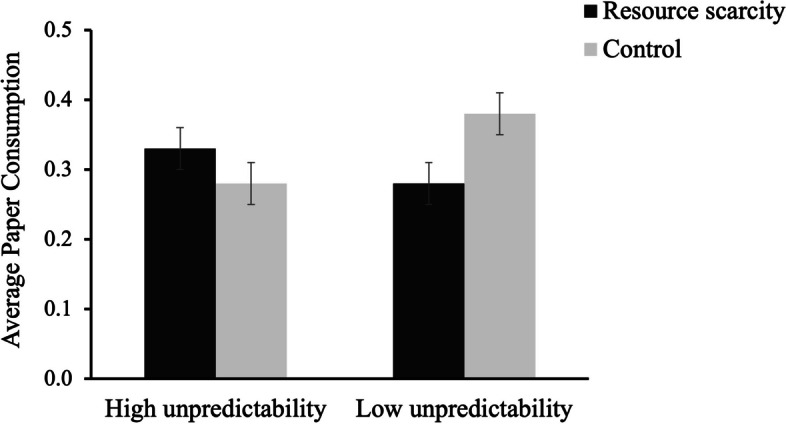
Average paper consumption as a function of resource scarcity and environmental unpredictability manipulations (Study 3)

Moreover, the results in average paper consumption showed a similar pattern. Main effects of scarcity (*F*(1,128) = 0.42, *p* > .05, 95%CI = [-0.137, 0.042]) and environmental unpredictability (*F*(1,128) = 0.70, *p* > .05, 95%CI = [-0.143, 0.036]) were not significant. A significant interaction effect was detected, *F*(1,128) = 5.30, *p* = .023, 95%CI = [0.021, 0.275], η_*p*_^2^ = .040. As predicted, in the *predictable* condition, paper consumption in the *scarcity* condition (*M* = 0.28, *SD* = 0.18) was significantly less than in the *control* condition (*M* = 0.38, *SD* = 0.18), *F*(1,128) = 4.39, *p* = .038, 95%CI = [-0.184, -0.005], η_*p*_^2^ = .033. No significant difference in paper consumption were observed between *scarcity* condition (*M* = 0.33, *SD* = 0.19) and *control* condition (*M* = 0.28, *SD* = 0.19) in *unpredictable* condition, *F*(1,128) = 1.39, *p* > .05, 95%CI = [-0.036, 0.143] (see Fig. 5).

More importantly, the above findings did not significantly differ without control variables in data analysis, and also did not significantly differ using the raw scores of water and paper consumption. Detailed results can be found in the Additional file 1.

**Fig. 6:**
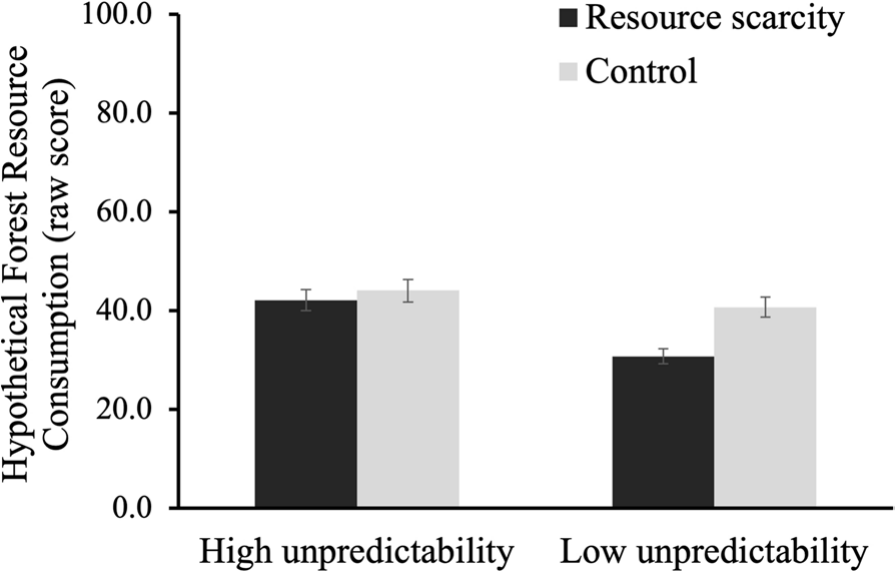
Forest resource consumption as a function of resource scarcity and environmental unpredictability manipulations (Study 3)

Hypothetical forest resource consumption was log transformed as it showed non-normal distribution. The findings showed that the main effects of scarcity (*F*(1,130) = 1.800, *p* > .05, 95%CI = [-0.363, 0.271]) and environmental unpredictability (*F*(1,130) = 2.189, *p* > .05, 95%CI = [-0.688, 0.049]) were not significant. A marginally significant interaction effect was detected, *F*(1, 130) = 3.04, *p* = .084, 95%CI = [-0.053, 0.849], η_*p*_^2^ = .023. As predicted, in the *predictable* condition, forest resource consumption in the *scarcity* condition (*M*_*raw*_ = 30.76, *SD*_*raw*_ = 14.97) was significantly less than the *control* condition (*M*_*raw*_ = 40.74, *SD*_*raw*_ = 25.41), *F*(1,130) = 4.71, *p* = .032, 95%CI = [-0.672, -0.031], η_*p*_^2^ = .035. No significant difference of forest resource consumption was observed between *scarcity* condition (*M*_*raw*_ = 42.15, *SD*_*raw*_ = 20.41) and *control* condition (*M*_*raw*_ = 44.00, *SD*_*raw*_ = 28.17) in *unpredictable* condition, *F*(1,130) = 0.08, *p* > .05, 95%CI = [-0.027, 0.363] (see Fig. 6).

### Brief discussion

As expected, Study 3 replicated the findings of the previous two studies. We identified a moderating effect of nature-caused environmental unpredictability on ecological resource scarcity information’s effect on actual PEB. Specifically, individuals who received lower levels of environmental unpredictability information exhibited more water-saving and paper-saving behaviors, and were inclined to harvest fewer forest resources in the face of water scarcity. Interestingly, even though our manipulation focused solely on water scarcity, both paper consumption and forest resource consumption were affected as well, despite their lack of direct association with water. These results highlight the robust influence of resource scarcity information and environmental unpredictability on PEB, thereby enhancing the ecological validity of our findings.

## General discussion

Focusing on the global issue of environmental unpredictability, the current research explored when does showing resource scarcity information promote PEB. In Study 1, a cross-sectional study, we discovered that resource scarcity information effectively enhances PEB, but only when individuals perceive the environment as less unpredictable. Furthermore, by manipulating scarcity information, Study 2 revealed that only for individuals with lower levels of environmental unpredictability could presenting ecological resource scarcity information decrease forest resource consumption intention. Moreover, an experiment with high ecological validity was conducted in Study 3 and found that the negative effect of water resource scarcity information on actual water and paper saving behaviors, as well as hypothetical forest resource consumption emerged only for people who receiving weaker environmental unpredictability information.

### Theoretical contribution and practical implication

Environmental unpredictability is an important concept in life history theory. Numerous studies have verified that childhood environmental unpredictability plays a crucial role in shaping life history strategies [27, 28, 30, 37, 53]. However, little is known about how adulthood environmental unpredictability functions. The current research provided preliminary evidence that unpredictability in adulthood can also function in shaping behaviors. Adulthood unpredictability, including both individual- and nature-related environmental unpredictability, demotivates individuals to sacrifice present interests for future environmental benefits when facing scarcity.

Some psychological factors, including those discussed earlier (such as short-sighted tendency, fear of free riders, and perceived lack of control), as well as self-interest and competitive orientation, can serve as potential mechanisms underlying the moderating effect of environmental unpredictability. Self-interest and competitive orientation are important ways for individuals to survive in a harsh environment. Individuals may adopt a competitive orientation to obtain more benefits for themselves to survive during periods of scarcity. In addition, they may also seek to weaken others’ interests. These factors have been identified as “Stone Age” psychological biases leading to environment destruction [54]. To better respond to ecological resource scarcity, the current research demonstrated the importance of creating a predictable and peaceful world by removing the psychological barriers to mitigate ecological resource scarcity.

The IMB model provides a comprehensive framework advancing resource conservation research and intervention implements [3]. Even though the IMB model captures three vital components, information, motivation, and behavioral skills on behavior change, the psychological barriers caused by environmental unpredictability were ignored. As illustrated in a recent meta-analysis [15], compared with the control group, of the 38 interventions including IMB components, water use was reduced by only 5.9% in average with a small effect size, and the magnitude of effect varied widely in different interventions. According to the findings in the current research, levels of environmental unpredictability may be the underlying reason for the varied efficacy. Therefore, to best strengthen reducing resource consumption interventions based on the IMB model, it’s necessary to take environmental unpredictability into consideration.

Importantly, the current research developed a new paradigm, washing-hands paradigm, to measure actual resource consumption in the lab. As illustrated in previous studies, there are gaps between self-reported behaviors, and objective behaviors [43]. However, over 80% of recent studies only relied on self-reported data [55]. The washing-hands paradigm sets up a situation to capture actual water and paper resource consumption data. Importantly, the confounding variables can be controlled in the paradigm, such as habits, individual difference on palm size, and social desirability. This paradigm can help to establish causality and improve ecological validity of lab experiments, advancing resource conservation research.

The current research also provides some vital practical implications for both policy makers and environmental organizations. Our data suggested that creating a predictable environment can help promote resource conservation when facing ecological resource scarcity information. Governments should try to eliminate unpredictable factors. However, some unpredictable factors are difficult to address, such as natural disasters and virus spread. In such conditions, individual-level practices appear to be more important. For countries with a predictable environment, the strategy of the reminders of the ecological resource scarcity information is effective. However, for countries with an unpredictable environment, governments and organizations can consider using public media to decrease residents’ perceived unpredictability. Moreover, inspired by our Study 2, emphasizing predictable environmental information when reminding residents of scarcity should be encouraged. Environmental organizations should provide information that the environment is predictable when calling for resource conservation to respond to scarcity.

### Limitations and future directions

The current research faces the limitation that the measurement in the correlation study is restricted due to the use of only one item to measure the moderator, and the alpha level of the PEB measure is low. For future studies, one aspect to consider is the exploration potential mechanisms of the moderation hypothesis. The current research did not delve into psychological mechanisms. It is suggested that future research could investigate underlying potential mechanisms of the moderation hypothesis to enrich the framework. Another related issue pertains to the IMB model. In the current research, we mainly focused on the effectiveness of scarcity information component but didn’t include motivation and behavioral skills components. It’s worthy for future research to test if creating a predictable environment can still strengthen the effect of IMB intervention. Besides, there are various types of resource conservation behaviors that individuals can engage in. Importantly, different behaviors are not necessarily highly relevant. For example, factors predicting shutting down electronics at night could not predict upgrading to energy-efficient appliances because these behaviors may cluster into distinct dimensions [56, 57]. In the current research, we may not be able to generalize our findings to other types of behaviors. Thus, future research is encouraged to investigate whether the moderation hypothesis can be verified in other types of resource conservation behaviors.

## Conclusion

Across three studies, we provided evidence to support the moderation hypothesis that environmental unpredictability weakens the positive effect of ecological resource scarcity information on PEB. Moving forward, it would be valuable to delve deeper into the underlying mechanisms, examine the moderation effect across various types of PEB, and investigate its potential application in PEB interventions.

### Supplementary Information

Supplementary Material 1.

## Data Availability

No datasets were generated or analysed during the current study.
