# Supplementary material for: Navigating an unpredictable environment: the moderating role of perceived environmental unpredictability in the effectiveness of ecological resource scarcity information on pro-environmental behavior
Source: BMC Psychol. 2024 May 10;12:261. doi: 10.1186/s40359-024-01762-1 (PMC11088101; doi:10.1186/s40359-024-01762-1)
Supplement: Supplementary file 1 — Supplementary Material 1. [file 40359_2024_1762_MOESM1_ESM.docx]

# Supplementary Materials for Navigating an Unpredictable Environment: The Moderating Role of Perceived Environmental Unpredictability in the Effectiveness of Ecological Resource Scarcity Information on Pro-Environmental Behavior

# Text S1. Additional Analyses of Study 2

We test the moderating effect on deforestation rate and number of acres cut separately.

Concerning deforestation rate, we regressed it on ecological resource scarcity information (dummy coded: 1 = *scarcity* condition, 0 = *control* condition), environmental unpredictability and their interaction by employing the PROCESS macro (Model 1, 5000 bootstrap samples) for SPSS Hayes (2013). The results showed a significant interaction effect (*b* = 0.27, *SE* = 0.11, *t* = 2.37, *p* = .019, 95%*CI* = [0.045, 0497], Δ*R*^2^ = .04), indicating that the effect of resource scarcity information on deforestation rate was moderated by environmental unpredictability. To be specific, for individuals with lower levels of environmental unpredictability (below 1 SD), participants in the *scarcity* condition tended to cut down trees slower relative to those in the *control* condition, *b* = -0.67, *SE* = 0.36, *t* = -1.85, *p* = .067, 95% CI = [-1.3877, 0.048]. In contrast, for individuals with higher levels of environmental unpredictability (above 1 SD), participants in the *scarcity* condition chose faster deforestation rate compared with those in the *control* condition, *b* = 0.64, *SE* = 0.37, *t* = 1.74, *p* = .084, 95% CI = [-0.088, 1.371] (see Figure S1). Moreover, a floodlight analysis was performed to decompose the interaction. It revealed that ecological resource scarcity manipulation decreased deforestation rate for any value of environmental unpredictability less than 0.66 (*b* = -0.83, *SE* = 0.42, *t* = -1.98, *p* = .05, 95% CI = [-1.613, 0.000]), while for any value greater than 7.01, scarcity manipulation would increase deforestation rate (*b* = 0.91, *SE* = 0.46, *t* = -1.98, *p* = .05, 95% CI = [0.000, 1.828]).


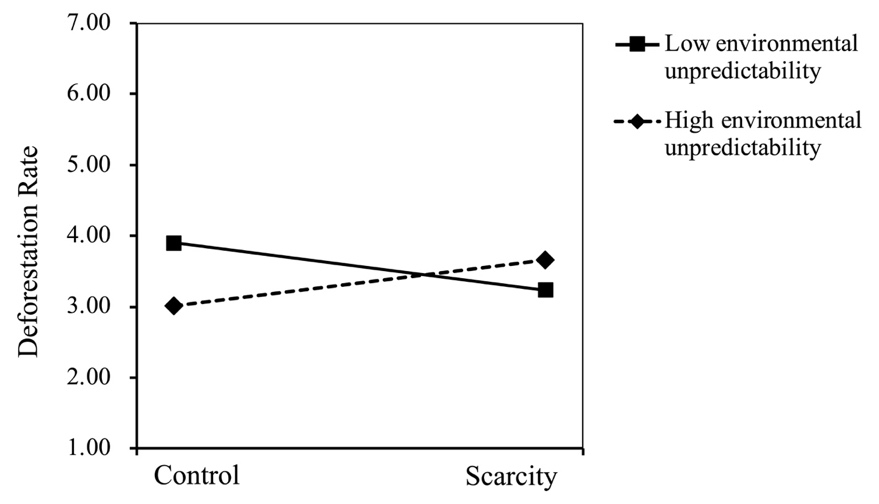


**Figure S1** The Effect of Ecological Resource Scarcity on Deforestation Rate at High and Low Levels of Environmental Unpredictability in Study 2.

As the distribution of the number of acres participants chose to cut was right-skewed (Skewness = .566, Kurtosis = -.329), we transformed the variable by taking the square root of the raw scores. Then PROCESS macro (Model 1, 5000 bootstrap samples) for SPSS Hayes (2013) was applied to test the moderating effect on the transformed number of acres cut. As predicted, the interaction effect was marginally significant, *b* = 0.27, *SE* = 0.14, *t* = 1.91, *p* = .058, 95%*CI* = [-.009, .542], Δ*R*^2^ = .02. Specifically, for individuals with lower levels of environmental unpredictability (below 1 SD), participants in the *scarcity* condition chose to cut less forest relative to those in the *control* condition, *b* = -1.09, *SE* = 0.41, *t* = -2.61, *p* = .0099, 95% CI = [-1.905, -.265]. In contrast, for individuals with higher levels of environmental unpredictability (above 1 SD), the ecological resource scarcity manipulation had no effect on number of acres cut, *b* = 0.07, *SE* = 0.43, *t* = 0.17, *p* > .05, 95% CI = [-.776, .924] (see Figure S2). Besides, the floodlight analysis revealed that ecological resource scarcity manipulation decreased forest resource consumption intention for any value of environmental unpredictability less than 3.32 (*b* = -0.58, *SE* = 0.30, *t* = -1.98, *p* = .05, 95% CI = [-1.168, 0.000]), but not for any value greater than 3.32. More importantly, the above findings did not significantly differ after controlling for demographic variables.


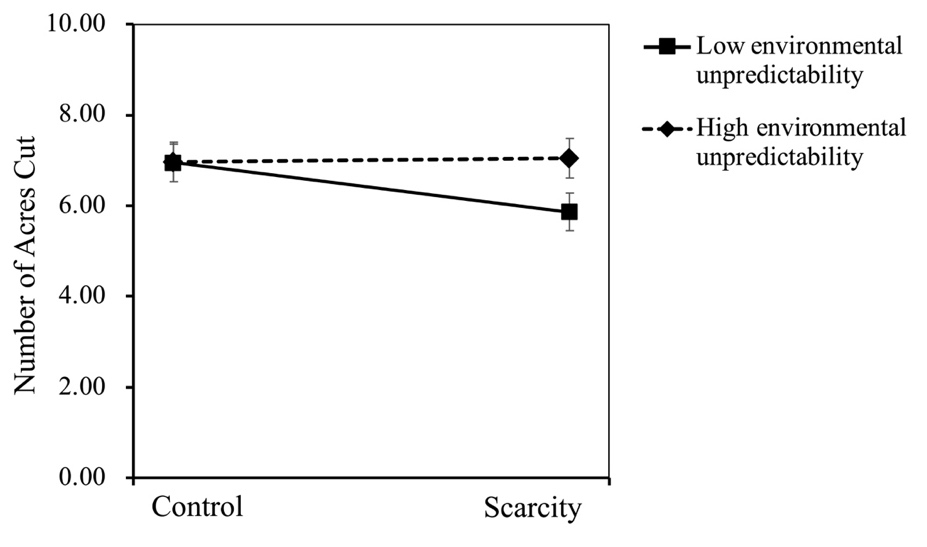


**Figure S2** The Effect of Ecological Resource Scarcity on Number of Acres Cut at High and Low Levels of Environmental Unpredictability in Study 2.

In general, the results indicated that environmental unpredictability weakened the negative effect of ecological resource scarcity information on both deforestation rate and number of acres participants intended to cut down, which was consistent with our moderating hypothesis.

# Text S2. Additional Analyses of Study 3

In Study 3, we included gender, age, and household income as control variables because that older people, females, and higher social-class individuals (higher educational level and higher income) were found to be more environmentally friendly behaved.^1^

For robustness check, we also test whether the results were consistent when removing the controls. Concerning the average water consumption, the findings revealed that the main effect of scarcity was significant (*F*(1,132) = 5.09, *p* = 0.026, 95%CI = [-14.449, -0.937], η*_p_*^2^ = .037), and the main effect of environmental unpredictability was not significant (*F*(1,132) = 0.48, *p* > .05, 95%CI = [-7.382, 6.138]). Consistently, the interaction was significant (*F*(1,132) = 6.32, *p* = .013, 95%CI = [2.586, 21.710], η*_p_*^2^ = .046). Then, simple effect analysis revealed that under the low unpredictability condition, the average water consumption was significantly less under the scarcity condition than under the control condition, *F*(1,132) = 11.36, *p* = .001, 95%CI = [-18.288, -4.763], η*_p_*^2^ = .079. However, under the high unpredictability condition, there was no significant difference between the scarcity condition and control condition, *F*(1,132) = 0.03, *p* > .05, 95%CI = [-6.138, 7.382].

Besides, main effects of scarcity (*F*(1,132) = 0.32, *p* > .05, 95%CI = [-0.154, 0.037]) and environmental unpredictability (*F*(1,132) = 0.61, *p* > .05, 95%CI = [-0.146, 0.044]) on average paper consumption were not significant. A significant interaction effect was detected, *F*(1,132) = 5.23, *p* = .024, 95%CI = [0.021, 0.289], η*_p_*^2^ = .038. Simple effect analysis showed that under the low unpredictability condition, the average paper consumption was significantly less under the scarcity condition than under the control condition, *F*(1,132) = 4.71, *p* = .032, 95%CI = [-.199, -.009], η*_p_*^2^ = .034. However, under the high unpredictability condition, there was no significant difference between the scarcity condition and control condition, *F*(1,132) = 1.13, *p* > .05, 95%CI = [-.044, .146].

Apart from this, we used the average water and paper consumption as the DVs because water and paper consumption for washing ink from hands might be affected by palm size. For robustness check, we further test whether the results did not significantly differ using the raw scores of water and paper consumption.

Specifically, for the water consumption, the main effect of scarcity was marginally significant (*F*(1,132) = 3.89, *p* = 0.051, 95%CI = [-1043.225, -43.156], η*_p_*^2^ = .029), and the main effect of environmental unpredictability was not significant (*F*(1,132) = 1.10, *p* > .05, 95%CI = [-503.348, 489.230]). And the interaction was significant (*F*(1,132) = 4.05, *p* = .046, 95%CI = [12.390, 1416.411], η*_p_*^2^ = .030). Then, simple effect analysis revealed that under the low unpredictability condition, the water consumption was significantly less under the scarcity condition (*M* = 1801.52, *SD* = 865.41) than under the control condition (*M* = 2508.86, *SD* = 1175.00), *F*(1,132) = 7.94, *p* = .006, 95%CI = [-1203.846, -210.838], η*_p_*^2^ = .057. However, under the high unpredictability condition, there was no significant difference between the scarcity condition (*M* = 2344.71, *SD* = 1134.43) and control condition (*M* = 2337.65, *SD* = 919.10), *F*(1,132) = 0.001, *p* > .05, 95%CI = [-489.238, 503.348].

Considering the effects on paper consumption, main effects of scarcity (*F*(1,132) = 0.56, *p* > .05, 95%CI = [-0.871, 0.400]) and environmental unpredictability (*F*(1,132) = 0.05, *p* > .05, 95%CI = [-0.995, 0.286]) were not significant. And the interaction effect was marginally significant, *F*(1,132) = 3.18, *p* = .077, 95%CI = [-.089, 1.709], η*_p_*^2^ = .024. To be specific, in the low unpredictability condition, paper consumption in the scarcity condition (*M* = 1.94, *SD* = 1.41) was marginally significantly less than in the control condition (*M* = 2.51, *SD* = 1.36), *F*(1,132) = 3.20, *p* = .076, 95%CI = [-1.211, 0.061], η*_p_*^2^ = .024. No significant difference in paper consumption were observed between scarcity condition (*M* = 2.29, *SD* = 1.34) and control condition (*M* = 2.06, *SD* = 1.18) in high unpredictability condition, *F*(1,132) = .54, *p* > .05, 95%CI = [-0.400, 0.871].

In general, our main findings did not significantly differ without control variables in data analysis, and also did not significantly differ using the raw scores of water and paper consumption.

# Reference

1. Gifford R, Nilsson A. Personal and social factors that influence pro-environmental concern and behaviour: A review: PERSONAL AND SOCIAL FACTORS THAT INFLUENCE PRO-ENVIRONMENTAL BEHAVIOUR. Int J Psychol. Published online 2014. doi:10.1002/ijop.12034
